# Decreased expression of the thyroid hormone-inactivating enzyme type 3 deiodinase is associated with lower survival rates in breast cancer

**DOI:** 10.1038/s41598-020-70892-4

**Published:** 2020-08-17

**Authors:** Iuri Martin Goemann, Vicente Rodrigues Marczyk, Mariana Recamonde-Mendoza, Simone Magagnin Wajner, Marcia Silveira Graudenz, Ana Luiza Maia

**Affiliations:** 1 Thyroid Unit, Endocrine Division, Hospital de Clínicas de Porto Alegre, Rua Ramiro Barcelos, 2350, Porto Alegre, RS CEP 90035-003 Brasil; 2 Institute of Informatics, Universidade Federal Do Rio Grande Do Sul, Porto Alegre, Brazil; 3 Bioinformatics Core, Hospital de Clínicas de Porto Alegre, Porto Alegre, Brazil; 4 Department of Pathology, Hospital de Clínicas de Porto Alegre, Porto Alegre, Brazil; 5 Faculdade de Medicina, Universidade Federal Do Rio Grande Do Sul, Porto Alegre, Brazil

**Keywords:** Proteins, Breast cancer, DNA methylation, Endocrine system and metabolic diseases, Breast cancer

## Abstract

Thyroid hormones (THs) are critical regulators of cellular processes, while changes in their levels impact all the hallmarks of cancer. Disturbed expression of type 3 deiodinase (DIO3), the main TH-inactivating enzyme, occurs in several human neoplasms and has been associated with adverse outcomes. Here, we investigated the patterns of DIO3 expression and its prognostic significance in breast cancer. DIO3 expression was evaluated by immunohistochemistry in a primary cohort of patients with breast cancer and validated in a second cohort using RNA sequencing data from the TCGA database. DNA methylation data were obtained from the same database. DIO3 expression was present in normal and tumoral breast tissue. Low levels of DIO3 expression were associated with increased mortality in the primary cohort. Accordingly, low *DIO3* mRNA levels were associated with an increased risk of death in a multivariate model in the validation cohort. DNA methylation analysis revealed that the *DIO3* gene promoter is hypermethylated in tumors when compared to normal tissue. In conclusion, DIO3 is expressed in normal and tumoral breast tissue, while decreased expression relates to poor overall survival in breast cancer patients. Finally, loss of DIO3 expression is associated with hypermethylation of the gene promoter and might have therapeutic implications.

## Introduction

Breast cancer is the most common cancer in women worldwide, accounting for more than two million new cancer cases and 14.9% of all cancer-related deaths in women in 2018^1^. Despite remarkable advances in the treatment of breast cancer in recent decades, not all patients benefit from current therapeutic options and thus will experience relapse^2,3^. Genomic tests improve the clinical prediction of patient outcomes and determine the necessity of adjuvant chemotherapy with endocrine therapy^3,4^. However, it is a highly heterogeneous disease that is diverse in its behavior and responsiveness to the different modalities of treatment^5,6^. Breast cancer is characterized based on receptor and gene expression profiles that, together with the classic clinicopathological variables, guide the treatment and estimate the risk of recurrence^3,4^. Gene expression profiling studies have established at least four molecularly distinct types of breast cancer that can be expanded to the “intrinsic” subtypes luminal A (LumA), luminal B (LumB), HER2-enriched, basal-like, and normal-like^7–9^.

Numerous studies have established thyroid hormones (THs) as critical regulators of multiple cellular processes in normal and tumor cells^10^. They contribute to cellular proliferation and differentiation during development and adulthood and are fine-tuned for tissue-specific control^10,11^. Clinical studies associate TH levels with breast cancer risk and mortality^12,13^, while in vitro models demonstrate the effect of THs on breast cancer cell proliferation, apoptosis, and migration^14–16^. T_4_ promotes cell proliferation through the αvβ3 integrin receptor^14^, while the proliferative effects of T_3_ depend, at least partially, on the presence of estrogen receptors in breast cancer cells^17,18^. Clinically, however, the effects of THs on specific histopathological and molecular subtypes of breast cancer are still unclear^19,20^.

Modulation of THs concentrations is orchestrated by a group of selenoproteins called iodothyronine deiodinases, which can activate and inactivate thyroid hormones^21^. Briefly, the type 1 deiodinase (DIO1) catalyzes both activation and inactivation of thyroxine (T_4_), generating triiodothyronine (T_3_) and reverse triiodothyronine (rT_3_), respectively^22^. Type 2 deiodinase (DIO2) acts locally, converting the prohormone T_4_ into the active T_3_. Meanwhile, type 3 deiodinase (DIO3) is the main TH-inactivating enzyme by degrading T_4_ and T_3_ to inactive metabolites (rT_3_ and diiodothyronine, respectively)^21^. The *DIO3* gene is found in the *DLK1-DIO3* genomic region, which is located on human chromosome 14q32^23^. *DIO3* gene is subject to genomic imprinting, an uncommon epigenetic phenomenon that results in the preferential expression of one of the alleles (paternal allele in the case)^24,25^. *DIO3* gene expression is increased in several tissues during embryogenesis, but it decreases in most tissues in adulthood^26,27^. Notably, DIO3 is expressed in normal and pathological hyperproliferative conditions, where it has been implicated in cell proliferation and differentiation^20,25,26,28^. In particular, studies have demonstrated that the local control of THs signaling provided by the regulation of DIO3 activity is associated with cancer development, progression, and recurrence^28–30^. We have previously reported that DIO3 mRNA and activity levels are increased in papillary thyroid cancer (PTC), which are associated with larger tumor size, and the presence of lymph node and distant metastasis at diagnosis^30^. Others have described hyperexpression of this enzyme in basal cell carcinoma (BCC), where it modulates intracellular T_3_ concentrations and thus contributes to the cell tumorigenic potential^31^. DIO3 exerts a similar function in colon cancer, which suggests that attenuation of the TH signal is part of the oncogenic process, at least in some types of cancer^28^.

Considering the implied role of the *DIO3* gene in human neoplasms and the potential effect of TH in breast carcinogenesis^13–15^, we investigated the expression patterns of *DIO3* in normal breast tissue and breast cancer. Here, we demonstrate that *DIO3* is expressed in normal breast tissue and breast cancer tissue. In breast cancer, reduced *DIO3* expression is associated with decreased overall survival. Interestingly, loss of *DIO3* expression might be explained, at least partially, by gene promoter hypermethylation.

## Results

### DIO3 in normal breast and fibroadenoma

**Figure 1:**
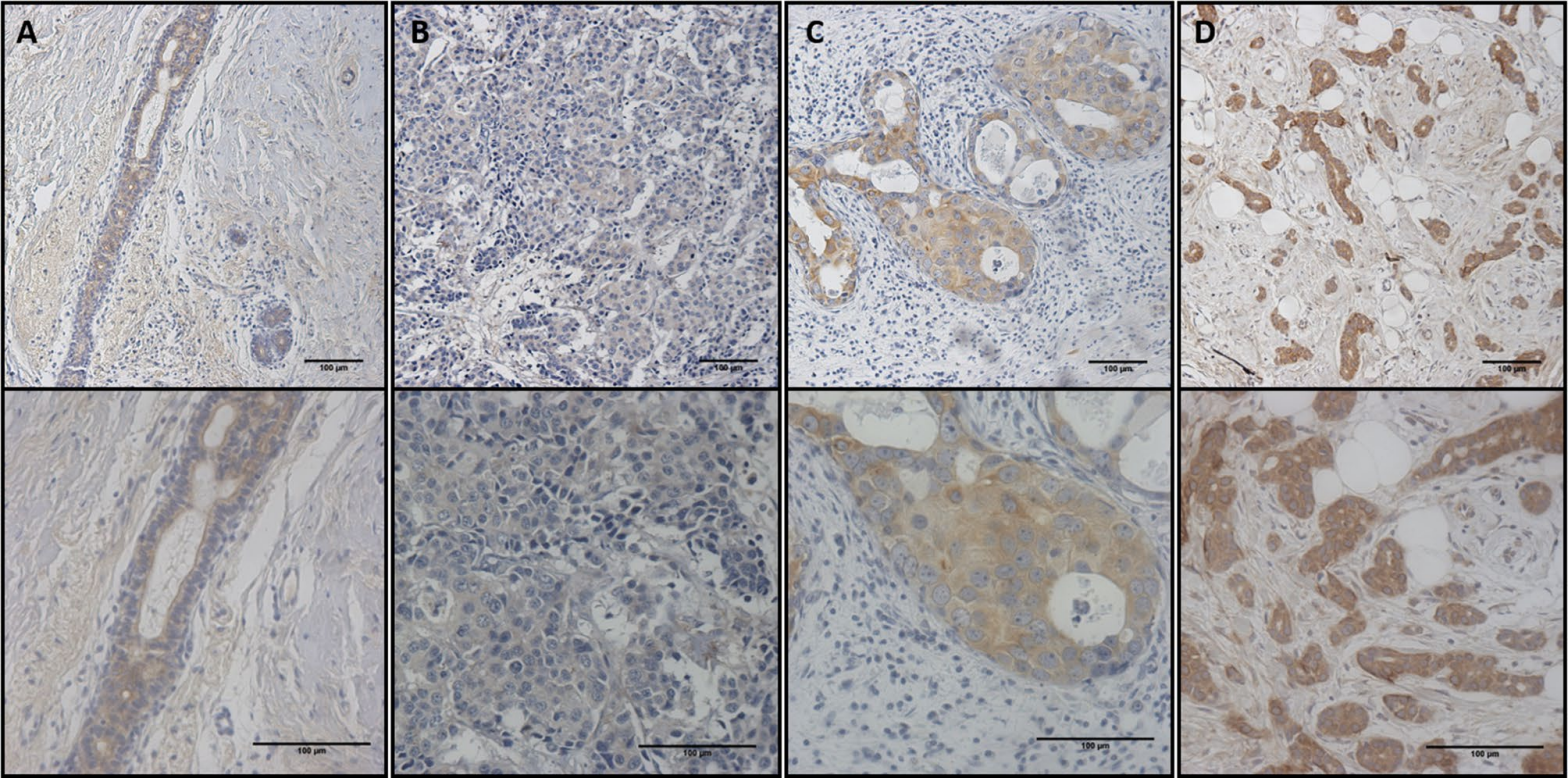
Patterns of expression of DIO3 in breast samples. Immunostaining was performed as described in Materials and Methods. From left to right: (**A**) normal glandular breast tissue, (**B**) breast carcinoma with low expression (overall intensity 1+), (**C**) breast carcinoma with moderate expression (overall intensity 2+) and (**D**) breast cancinoma with high expression (overall intensity 3+) of DIO3 protein evaluated through immunohistochemistry. The staining intensity level is used to calculate the H-score, combined with the percentage of positive cells (see “Methods”).

DIO3 immunohistochemistry staining was detected in all samples of normal breast tissue (N = 5) at an overall moderate intensity (H-score = 160 ± 63). DIO3 staining was predominantly cytoplasmatic and more pronounced in the apical extremity in luminal cells in both ducts and acini of the breast (Fig. 1A). DIO3 was markedly positive in myoepithelial cells (Fig. 1A, bottom). Benign fibroadenoma lesions (N = 4) were also positive for DIO3 staining, with an intensity comparable to healthy tissue (H-score = 153 ± 41 vs. 160 ± 63, *P* = 0.75).

### DIO3 protein in breast cancer: the primary cohort

To study DIO3 expression in breast cancer, we analyzed a cohort of patients who had been seen at our institution (primary cohort, N = 44) and validated the results in the TCGA-BRCA cohort (validation cohort, N = 1,094). The clinicopathological characteristics of the patients from both cohorts are summarized in Table 1.

**Table 1:** Baseline characteristics of patients with breast cancer included in the primary cohort and in the validation cohort.

Characteristic	Primary cohort (N = 44)	Validation cohort (N = 1,094)
Median age at diagnosis (range)—years	52 (26–71)	59 (26–90)
**Tumor size in the largest dimension—mm**		
Median (IQR)	20 (13–30)	N/A
Mean (± SD)	31.15 ± 29.1	N/A
**Estrogen receptor—no (%)**		
Positive	25 (58.1%)	807 (73.7%)
Negative	18 (41.9%)	237 (21.6%)
Missing	0	50 (4.6%)
**Progesterone receptor—no (%)**		
Positive	24 (55.8%)	698 (63.8%)
Negative	19 (44.2%)	343 (31.3%)
Missing	0	53 (4.8%)
**HER2 status—no (%)**		
Positive	12 (27.9%)	114 (13.2%)
Negative	30 (69.8%)	649 (59%)
Missing	1 (2.3)	331 (30%)
**Histological type of tumor—no (%)**		
Invasive Ductal Carcinoma (IDC)	40 (90.9%)	813 (79.7%)
Invasive Lobular Carcinoma (ILC)	3 (6.8%)	207 (20.3%)
Ductal Carcinoma in situ (DCIS)	1 (2.3%)	0
**Clinical-pathological subtype—no (%)**	AJCC* 2018	PAM50**
Luminal A	8/44 (18.2%)	231 (45%)
Luminal B	17/44 (38.6%)	127 (24.7%)
HER2	7/44 (15.9%)	58 (11.3%)
Triple Negative	10/44 (22.7%)	97 (18.9%)
Non classified	2 (4.5%)	
**Lymph node metastasis—no (%)**		
Yes	17 (39%)	558 (52%)
No	26 (61%)	516 (48%)
**Distant metastasis—no (%)**		
Yes	4 (9.3%)	14 (1.8%)
No	39 (91.7%)	768 (98.2%)
**Tumor staging—no (%)**		
Stage I/II	30/44 (68.2%)	182 (73.2%)
Stage III/IV	12/44 (27.3%)	269 (24.6%)
Missing	2 (4.5%)	24 (2.2%)
**Pre-treatment hypothyroidism—no (%)**	1/43 (2.3%)	N/A
**Post-treatment hypothyroidism—no (%)**	3/43 (7%)	N/A
**Follow-up (mean ± SD)—months**	81.9 +—32.7	22.2 (12.9–47.5)
**All-cause mortality—no (%)**	11/43 (25.5%)	152/1,094 (13.9%)
**Mean survival months (95% CI)**	115.7 (102.2–129.2)	153.7 (136.8–170.6)

*N/A* not available, *IQR* interquatile range, *SD* standard deviation, *HER2* human epidermal growth factor receptor2, *AJCC* American Joint Committee on Cancer.

*Classified by the AJCC 2018 staging system.

**Classified by PAM50, data available for 513 patients.

**Figure 2:**
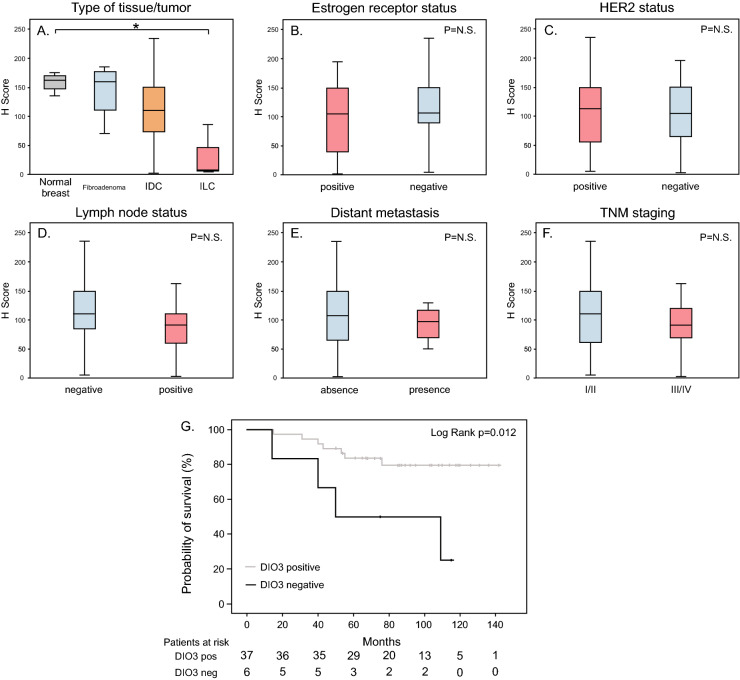
DIO3 staining and clinicopathological characteristics of patients with breast cancer in the primary cohort. (**A**–**F**) Box plots of DIO3 staining in breast tissue samples evaluated through immunohistochemistry and quantified by H-Score. Samples were divided according to clinicopathological data as follows: (**A**) type of tissue analyzed, (**B**) ER status, (**C**) HER2 status, (**D**) lymph node status, (**E**) distant metastasis and (**F**) TNM anatomic staging. (**G**) Kaplan–Meier plot of overall survival in patients with the presence (gray) or absence (black) of DIO3 staining in breast cancer evaluated through immunohistochemistry.* ER* estrogen receptor,* HER2* human epidermal growth factor receptor2,* IDC* invasive ductal carcinoma, *ILC* invasive lobular carcinoma,* N.S.* not significant *P = 0.05.

Patterns of DIO3 staining evaluated through immunohistochemistry in breast cancer samples are shown in Fig. 1B–D. DIO3 staining in FFPE breast cancer tissues was positive in 35/39 (89.7%) samples of invasive ductal carcinoma (IDC), with a mean H-score of 104.9 ± 55. When evaluating invasive lobular carcinoma (ILC), only 1 of 3 samples was positive for DIO3 (H-score = 86). A sample of ductal carcinoma in situ (DCIS) was also positive for DIO3 expression (H-score = 100). A graph comparing the H-score for DIO3 in non-malignant tissues and malignant breast cancer types is presented in Fig. 2A. Mean DIO3 H-scores of primary tumors were similar to the non-tumoral tissues, with a marginal decrease in DIO3 seen in invasive lobular carcinoma (ILC) (*P* = 0.05). The mean H-score of invasive ductal carcinoma was similar to that of normal tissue (*P* = 0.78). No differences were observed between the molecular subtypes of breast cancer (*P* = 0.8) (data not shown). There was no difference in the H-score between tumors with ER-positive and ER-negative status (*P* = 0.31) (Fig. 2B) or between tumors with HER2-positive and HER2-negative status (*P* = 0.81) (Fig. 2C). Among the primary tumors, there was no significant correlation between H-score and Ki-67(%) levels (*P* = 0.9), or between H-score and histological tumor grade (*P* = 0.43).

We found no association of DIO3 positivity (negative or positive) with tumor size (*P* = 0.18). The mean H-score in primary tumors of patients without nodal metastases was similar to that observed in patients with lymph node metastasis (*P* = 0.07). Similarly, H-scores of primary tumors of patients with distant metastasis did no differ from those without distant metastasis (*P* = 0.78; Fig. 2D,E). There were no differences on DIO3 H-scores when comparing patients with stage I/II vs. stage III/IV disease (*P* = 0.41) (Fig. 2F). We obtained both primary and lymph node tissues from 5 patients. In this subset of patients, DIO3 staining was comparable between paired primary tumor and lymph node metastasis (*P* = 0.36).

Table 2 shows the variables associated with an increased risk of death in the primary cohort (univariate analysis). We observed that negative DIO3 staining was associated with poor prognosis (HR 4.29; 95% CI 1.24 to 14.7; *P* = 0.021). Therefore, additional studies were performed using Kaplan–Meier analysis and the log-rank test. Patients with negative DIO3 staining had a worse overall survival than those with positive DIO3 staining. The mean overall survival was 73.3 months (95% CI 41 to 105) in the DIO3-negative group and 122 months (95% CI 109 to 135) in the DIO3-positive group (Fig. 2G, log-rank *P* = 0.012).

**Table 2:** Univariate Cox regression analysis of overall survival in breast cancer patients in the primary cohort.

Variable	HR (95% CI)	*P* value
Age at diagnosis (years)	1.01 (0.95–1.06)	0.74
Tumor size (mm)	1.03 (1.01–1.04)	0.002
Lymph node metastasis (pos vs. neg)	4.71 (1.24–17.81)	0.026
Distant metastasis (pos vs. neg)	4.57 (1.17–17.77)	0.029
ER status (pos vs. neg)	0.54 (0.16.–1.79)	0.32
P status (pos vs. neg)	0.40 (0.12–1.38)	0.15
HER2 positivity (pos vs. neg)	1.80 (0.49–6.42)	0.38
TNM staging (III/IV vs I/II)	6.54 (1.83–23)	0.003
DIO3 status (neg vs. pos)	4.29 (1.24–14.7)	0.021

*HR* hazard ratio, *CI* confidence interval, *ER* estrogen receptor, *P* progesterone, *HER2* human epidermal growth factor receptor2.

### *DIO3* mRNA in breast cancer patients: validation cohort

**Figure 3:**
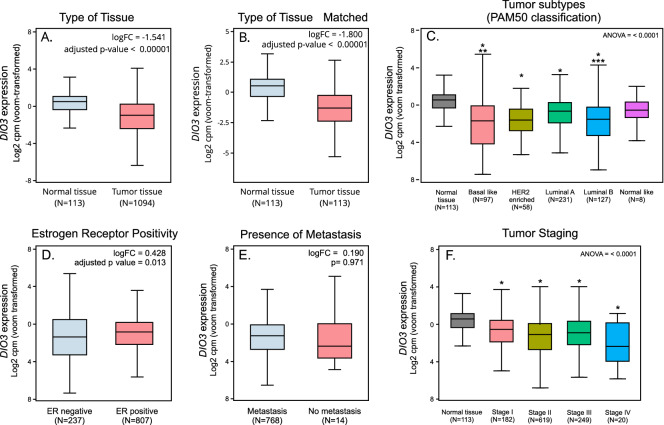
The relationship between *DIO3* mRNA expression and clinicopathological parameters in breast cancer samples of patients from the TCGA-BRCA cohort expressed in Log2 counts per million (*voom*-transformed). Comparative expression demonstrates that *DIO3* mRNA is decreased in tumoral tissue when compared to normal tissue when analyzing (**A**) all samples or (**B**) only matched samples. (**C**) All tumor subtypes have decreased expression of *DIO3* mRNA when compared to normal tissue, with the exception of normal-like tumors compared to normal tissue. *DIO3* mRNA levels were also reduced in basal-like tumors when compared to luminal A (**) (logFC = -1.024; adjusted *P* value = 0.0003) and in luminal B when compared to luminal A subtypes (***) (logFC = -0.915 adjusted *P* value = 0.0009), and (**D**) *DIO3* expression is increased in ER-positive samples when compared to ER-negative samples. (**E**) *DIO3* expression is similar in patients with or without metastasis. (**F**) When samples were separated according to tumor staging, all tumor stages had decreased *DIO3* expression when compared to normal tissue, but there was no difference in expression between the stages. *ER* estrogen receptor. *Adjusted *P* value < 0.0001 in comparison to normal tissue.

It has been previously demonstrated that DIO3 protein levels and activity correlate with *DIO3* mRNA levels in different contexts^30,32,33^. Therefore, to validate differences of DIO3 expression among patients with breast cancer, we analyzed *DIO3* mRNA expression in a second cohort using available gene expression data from the TCGA-BRCA study. In this second population, *DIO3* expression was found to be reduced in primary solid tumors (N = 1,094) compared to that observed in normal breast samples (N = 113, logFC = -1.54, adjusted *P* value < 0.00001, Fig. 3A), even when the comparison was made only with matched normal tissues (logFC = -1.800 adjusted *P* value < 0.00001, Fig. 3B). The majority of tumor subtypes (with the exception of normal-like tumors), classified according to PAM50 classification system, showed reduced *DIO3* expression compared to normal tissue (Fig. 3C). On the other hand, *DIO3* expression was increased in ER-positive samples compared to that in ER-negative samples (logFC = 0.428; *P* = 0.013, Fig. 3D). There was no significant difference when comparing *DIO3* expression between patients with or without lymph node disease (logFC = 0.0359, adjusted *P* value = 0.914) or distant metastasis (logFC = -0.190, adjusted *P* value = 0.971, Fig. 3E). Decreased *DIO3* mRNA expression was observed in all tumor stages compared to that seen in normal tissue (*P* < 0.01). However, no differences were found between the different tumor stages (Fig. 3F). Interestingly, lower *DIO3* expression was associated with greater tumor size (*P* = 0.019) and ER negativity (*P* = 0.022).

**Figure 4:**
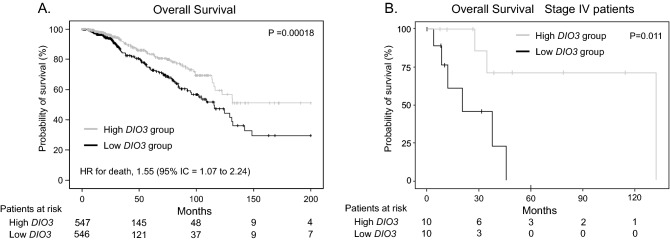
Kaplan–Meier estimates of overall survival in patients of the TCGA-BRCA cohort according to *DIO3* mRNA expression. Patients were grouped according to the median of DIO3 expression in the population as presenting high DIO3 expression (gray lines) or low DIO3 expression (black lines). Plot A shows the overall survival in the entire cohort. Plot B refers only to patients with stage IV disease. HR = hazard ratio; CI = confidence interval.

We then evaluated the prognostic value of *DIO3* mRNA expression for patient survival. We considered patients as having high *DIO3* expression when their logCPM values were above the median and as having low *DIO3* expression when their logCPM values were below the median. Low *DIO3* expression was associated with reduced survival, with an HR of 1.60 (95% CI 1.18 to 2.26; *P* = 0.003) in the univariate model (Table 3). Additional analysis using a multivariate model adjusted for all variables with a *P* < 0.1 in the univariate analysis demonstrated that low *DIO3* was an independent prognostic factor for death (HR 1.55; 95% IC 1.07 to 2.24; *P* = 0.02; Table 3, Fig. 4A). The estimated overall survival rate at five years in the Kaplan–Meier analysis was 90.4% (95% CI, 86.4% to 94.5%) in the high *DIO3* group and 77.4% (95% CI, 71.3% to 84.1%) in the low *DIO3* group (Fig. 4A).

**Table 3:** Univariate and multivariate Cox regression and for overall survival in the validation cohort.

Variables	Univariate analysis		Multivariate analysis*	
	HR (95% CI)	*P* value	HR (95% CI)	*P* value
Age at diagnosis (years)	1.03 (1.02–1.04)	< 0.001	1.04 (1.02–1.05)	< 0.001
Tumor size (≥ 2 cm vs ≤ 2 cm)	1.48 (1.00–2.18)	0.045	1.31 (0.83–2.08)	0.25
Lymph node (pos vs. neg)	2.13 (1.49–3.05)	< 0.001	1.87 (1.24–2.81)	0.003
Distant metastasis (pos vs. neg)	4.33 (2.57–7.20)	< 0.001	2.92 (1.61–5.30)	< 0.001
E2 status (pos vs. neg)	0.71(0.48–1.00)	0.056	0.66 (0.36–1.22)	0.187
P status (pos vs. neg)	0.31 (0.52–1.02)	0.066	0.31 (0.42–1.31)	0.309
HER2 positivity (pos vs. neg)	1.43 (0.89–2.28)	0.13		
TNM staging (III/IV vs I/II)	2.49 (1.78–3.48)	< 0.001		
DIO3 status (low vs. high)	1.60 (1.18–2.26)	0.003	1.55 (1.07–2.24)	0.02

*HR* hazard ratio, *CI* confidence interval, *ER* estrogen receptor, *P* progesterone, *HER2* human epidermal growth factor receptor2.

*All variables with *P* < 0.1 were included in the multivariate model. TNM is not included as it is derived from variables already present in the model.

In the subgroup analysis of patients with advanced disease (stage IV), those with low *DIO3* expression had reduced overall survival compared to patients with high *DIO3* expression (*P* = 0.011; Fig. 4B). Notably, low *DIO3* expression was associated with worse overall survival among patients with ER-positive tumors (*P* = 0.0012) but not among those with ER-negative tumors (*P* = 0.89) (Supplementary Fig. 1).

### Methylation of *DIO3* gene promoter

**Figure 5:**
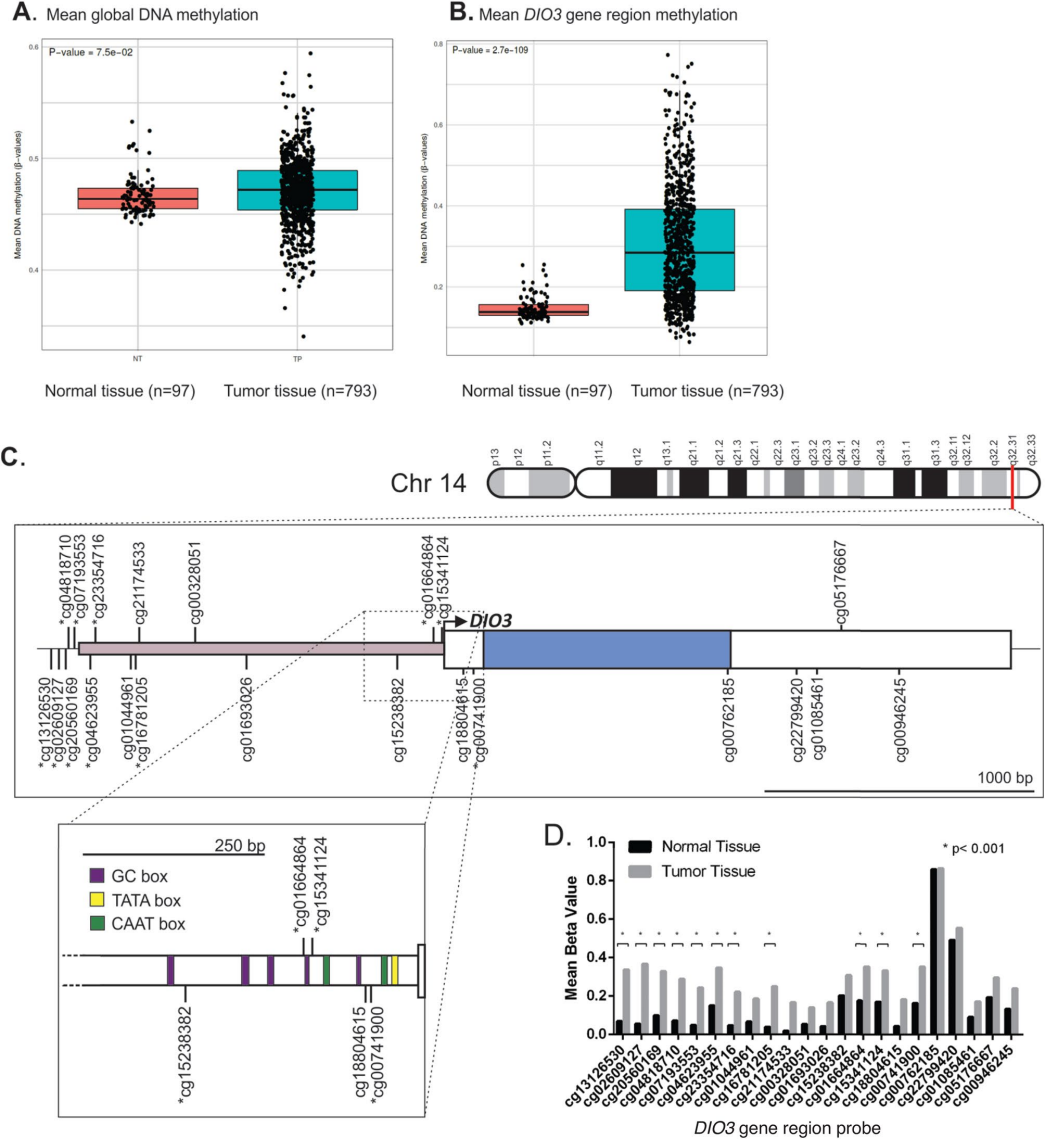
Panel (**A**) demonstrates mean global DNA methylation levels (β-values) in breast cancer tissue compared to healthy breast tissue. Panel (**B**) demonstrates that the mean DNA methylation of *DIO3* gene region is increased in tumor tissue when compared to normal tissue (*P* < 0.001). Panel (**C**) is a schematic representation of the location of *DIO3* gene in chromosome 13 and the regions that were evaluated by CpG probes. The promoter region is composed by several promoter elements including a TATA box, two CAAT boxes and CG rich regions (**C**, enhanced). Significant hypermethylation in several CpG sites (*) is observed in the promoter region of the gene. Panel (**D**) presents mean β-values of CpG sites mapped in *DIO3* gene region comparing normal and tumoral tissue.

To further investigate possible factors that could lead to decreased *DIO3* expression in breast cancer, we performed DNA methylation analysis of a subgroup of patients from TCGA-BRCA database from whom DNA methylation data were available (N = 890). Our analysis demonstrated that global DNA methylation levels of breast cancer samples were similar to those of healthy breast tissues (Fig. 5A). However, the methylation levels of CpG sites in the *DIO3* gene region were increased compared to those from healthy tissue (Fig. 5B) (*P* < 0.0001). Figure 5 details the CpG sites that are hypermethylated (*) within the *DIO3* gene region. The first 1.5 kbp of 5′ flanking region (red) are known to be extremely G + C rich (80% of the sequence), and this region is highly conserved between mouse and human genome^34^. Promoter region (~ -250 bp of the 5′ flanking region) is composed of several promoter elements (Fig. 5C, enhanced), including a TATA box, two CAAT boxes and CG rich regions^35^. We observed a significant increase in DNA methylation levels in CpG sites that are located both at the promoter region and in the 5′ flanking 1.5 kbp conserved region of the gene (Fig. 5C,D).

## Discussion

Disruption of the iodothyronine deiodinases expression leads to changes in TH concentrations, which might contribute to cancer development and progression by impacting virtually all the hallmarks of cancer^10^. Here, we demonstrate that the TH-inactivating enzyme DIO3 is expressed in normal breast tissue and that its expression is highly prevalent in breast cancer. More interestingly, our results demonstrated that low DIO3 expression was an independent prognostic factor for reduced overall survival in two different populations of patients with breast cancer.

Data on the expression of iodothyronine deiodinases in human breast tissue are scarce. Low levels of DIO1 were reported in normal and lactating tissues, but DIO2 and DIO3 have not been analyzed thus far^36^. Here we show that DIO3 is expressed at both the mRNA and protein levels in normal human breast tissue. Expression of *DIO3* mRNA has been previously described in breast cancer cell lines (MCF-7 and MDA-MB-231 cells). *DIO3* mRNA was found to be upregulated in MCF-7 cells and down-regulated in MDA-MB-231 cells when compared to the non-tumoral cell line (MCF-10A cells). DIO3-mediated T_3_ deiodination also occurs in MCF-7 cells. In these cells, DIO3 expression is to regulated by retinoids but not by estradiol^37–39^. These findings are consistent with the presence of DIO3 in other tissues of ectodermal origin, such as the skin and the nervous system^40,41^.

The role of thyroid hormone metabolism on human tumorigenesis has been largely debated^10^. In breast cancer, previous studies showed that higher levels of the thyroid hormone receptor alpha were an independent prognostic factor for increased overall survival^42^. More recently, high levels of the thyroid hormone receptor beta in breast tumors were also associated with increased breast cancer-specific survival^43^.

In basal cell carcinomas (BCC), for instance, a DIO3-mediated decrease in T3 levels relates to increased cell proliferation^31^. Similarly, in colon cancer cells, DIO3 knockdown and consequent increases in T3 levels are associated with reduced cell proliferation and induction of differentiation^44^. High levels of DIO3 expression in primary PTC tumors were associated with advanced disease at the diagnosis^30^. Some data indeed suggest that T3 can contribute to tumor growth in breast cancer cells in vitro^17^, while a microenvironment with low T3 levels could facilitate invasiveness and dedifferentiation. However, in agreement with our data in breast cancer, similar levels of DIO3 mRNA are observed in glioblastoma and liver carcinomas as compared to respective normal tissues^45^. These differences could be attributed to the tissue embryological origin since the tissues of ectodermal origin seem to maintain DIO3 expression during adulthood, while DIO3 gene is subject to imprinting in other tissues. Loss of DIO3 expression was associated with tumor aggressiveness in colon cancer and also in thyroid cancer. DIO3 expression is present in papillary and follicular subtypes, but not in the most aggressive and dedifferentiated anaplastic subtype^30^. Taken together, these results indicate that, although expression of the enzyme is often upregulated in the neoplastic tissue compared to normal tissue, loss of DIO3 expression is a common hallmark of dedifferentiation in the neoplastic process, which might confer its prognostic significance. Alternatively, the distinct pattern of expression could be the result of DIO3 regulation or related to the cancer-type specific methylation signature.

Although this was an exploratory study, our results point to a prognostic role for DIO3 expression in breast cancer. In a primary cohort of 44 patients with breast cancer, negative DIO3 staining in the primary tumor was associated with significantly worse prognosis (HR 4.29; 95% CI 1.24 to 14.7; *P* = 0.021) when compared to patients who were DIO3-positive. More interesting, in the second cohort, low *DIO3* expression was an independent prognostic factor for death in a model adjusted for age, tumor size, lymph node and distant metastasis, estrogen and progesterone status (HR 1.55; 95% IC 1.07–2.24; *P* = 0.02). The prognostic role of *DIO3* expression was particularly relevant in the subgroup of patients with advanced disease.

Intriguingly, the difference in survival between groups with distinct DIO3 expression was limited to ER-positive patients. Previous studies indicate the existence of a crosstalk between estrogen- and TH-dependent regulatory pathways in breast cancer^14,17,46,47^, which might be a potential explanation. T_3_ regulates cell cycle progression and proliferation in breast cancer cells in vitro by a common mechanism involving ER and T_3_ receptor-mediated pathways^46^. Moreover, T_4_ can phosphorylate nuclear ER-alpha in MCF-7 cells via a MAPK-dependent pathway, promoting proliferation^14^. Therefore, loss of DIO3 expression and the consequent increase in intracellular T_3_ levels could be specifically detrimental to tumors that express ER, as our results suggest. Contributing to this interplay, previous studies have demonstrated that estrogen, progesterone and their receptors regulate DIO3 activity in rat uteri and decidua^48,49^. Therefore, we cannot rule out that in the breast, DIO3 expression depends partially on the presence of functional estrogen and progesterone receptors.

The *DIO3* gene is subject to genomic imprinting, an uncommon epigenetic phenomenon that results in the preferential expression of one allele (the paternal allele, in this case)^24,25^. The disturbed expression of genes and miRNAs, or altered hypermethylation patterns of the *DLK1-DIO3* genomic region, is involved in the pathogenesis of different types of cancer^50–53^. Thus, we hypothesized that the loss of *DIO3* expression in breast tumors could be a consequence of gene hypermethylation in the tumoral context. Indeed, our results show that while the mean global methylation in breast tumors is comparable to that of normal tissue, the *DIO3* genomic region, especially its promoter region, is significantly hypermethylated in tumors (Fig. 5C, enhanced). These findings might explain, at least in part, the reduced DIO3 expression in breast cancer. Of interest, the *DIO3* gene was also found to be hypermethylated in B-cell, T-cell and myeloid malignancies, and lung cancer^51,52^.

Our study has some limitations. The absence of data on DIO3 enzymatic activity limits the assumption that the decreases of DIO3 levels cause alterations in intracellular TH homeostasis. Alternatively, changes in DIO3 expression could simply represent a consequence of broader epigenetic modifications in the tumoral context. It is also important to consider that complete clinical data on patient thyroid status was not available, which could interfere with deiodinase expression^54, 55^. Therefore, the complex changes on deiodinases and the overall effect on intracellular TH status are still unclear in breast cancer. Additionally, our analysis is limited to two populations, using two different methodology, and, despite robust supporting data, results should be confirmed in other cohorts.

In conclusion, the results of this study demonstrate *DIO3* expression in breast tissue and breast cancer. Importantly, low *DIO3* expression is associated with reduced overall survival, suggesting that DIO3 might have a prognostic role in this disease. Reduced *DIO3* expression in breast cancer can be explained at least in part by gene hypermethylation. Due to its potential to modulate thyroid hormone intracellular levels and interplay with estrogen metabolism in breast cancer, the *DIO3* expression might have therapeutic implications.

## Methods

### Patients and tissues: primary cohort

Neoplastic tissue from 44 patients diagnosed with breast cancer was retrospectively collected from a consecutive series of unselected patients in the pathology department of Hospital de Clínicas de Porto Alegre. Tissue samples of the normal breast (N = 5) and fibroadenomas (N = 4) were also obtained. Histopathological reports containing information on tumor type, grade and immunohistochemistry were retrieved; clinical data were retrospectively reviewed in medical records. Tumors were histologically classified according to the 8th edition of the American Joint Committee on Cancer (AJCC) staging system^56^. All procedures performed in studies involving human participants were in accordance with the ethical standards of the institutional and/or national research committee. The study was reviewed and approved by the Institutional Review Board and Research Ethics Committee from the Hospital de Clínicas de Porto Alegre with a waiver of informed consent (Protocol number 16-0246).

### Immunohistochemistry studies and DIO3 staining assessment

DIO3 protein expression was evaluated by immunohistochemical studies on 6-mm sections of formalin-fixed paraffin-embedded (FFPE) tissue blocks from normal breast tissues, fibroadenomas, and primary breast cancers. The immunohistochemical technique consists of tissue deparaffinization and rehydration, antigenic recovery, inactivation of endogenous peroxidase and blockage of unspecific reactions. Samples were incubated overnight at a temperature of 4 °C with an anti-DIO3 rabbit polyclonal antibody (Abcam 102926, Cambridge, UK) at a dilution of 1:50, followed by subsequent incubation with a biotinylated secondary antibody, a streptavidin–HRP conjugate (LSAB; Dako, Carpinteria, CA, USA) and diaminobenzidine tetrahydrochloride (Kit DAB; Dako). The slides were examined using an Olympus BX51 microscope. The QCapture Pro software (Qimaging, Surrey, BC, Canada) was used to capture the images. DIO3 staining was evaluated by an experienced pathologist blinded to the molecular profile and TNM staging. The immunohistochemical results of DIO3 staining were assessed dichotomously (negative or positive) and semiquantitatively using the H-score method as described previously^57,58^. The H-score combines the percentage of positive cells and staining intensity level (weak 1 + , moderate 2 + , strong 3 +) and is calculated using the following formula: [1 × (% cells 1 +) + 2 × (% cells 2 +) + 3 × (% cells 3 +)], with results ranging from 0 to 300. Positive (epidermis and placenta, and epidermal nevus) and negative (connective and adipose tissue) internal controls were assessed for all the evaluated cases. Samples from the primary cohort were classified concerning the presence or absence of these receptors and the level of Ki-67 expression into the following groups: Luminal A (LumA), luminal B (LumB), triple negative and HER2. A Ki-67 index cut point of 14% was defined to distinguish HER2 negative lumB from lumA tumors^59,60^.

### Differential gene expression and methylation analysis

For the validation cohort, RNA sequencing (RNA-Seq) RSEM gene expression data from The Cancer Genome Atlas (TCGA) breast cancer (BRCA) study were obtained from the Genomic Data Commons (GDC) Data Portal (https://gdc-portal.cni.nih.gov) using the TCGAbiolinks R/Bioconductor package^61^. Raw expression signals for primary solid tumor samples (N = 1,094) and solid normal tissue samples (N = 113) were normalized and analyzed for differential expression of *DIO3* using the limma-voom pipeline from the limma R/Bioconductor package^62^. *P* values were adjusted for multiple comparisons using the false discovery rate (FDR) procedure of Benjamini and Hochberg^63^. Clinicopathological information for TCGA-BRCA samples was downloaded through TCGAbiolinks and the Broad GDAC Firehose (https://gdac.breadinstitute.org) (merged level 1 clinical data). For tumors of the TCGA-BRCA cohort, data retrieved from PAM50 classification were used to define tumor subtype classification^64^. Overall survival (OS) was estimated by the Kaplan–Meier method and compared by the log-rank test using functions provided by TCGABiolinks. For the methylation analysis, we used the TCGAbiolinks R/Bioconductor package^30^ to obtain and analyze Illumina 450 K methylation and clinical data for 890 samples from the TCGA-BRCA study, including 97 samples from healthy tissues and 793 from primary solid tumors. Differentially methylated CpG sites for *DIO3* were screened with the TCGAanalyze_DMR function, adopting an FDR-adjusted Wilcoxon rank-sum *P* value < 0.05 and a minimum absolute difference among group beta values (Δ *beta*) of 0.15.

### Statistical analysis

Clinicopathological data were reported as the mean and standard deviation or the median with 25th and 75th percentiles for the continuous variables, and frequency and percentages were reported for categorical variables. Student’s t-test or chi-square tests were used to compare clinicopathological variables, and Student’s t-test or one-way ANOVA was used to compare H-scores between different groups. A Cox proportional hazard model was used to test the univariable and multivariable statistical effects of DIO3 protein and *DIO3* mRNA expression on patient survival. Time-to-event analysis was performed with overall survival as the primary outcome and was evaluated with log-rank analysis using Kaplan–Meier curves and both unadjusted and multivariable Cox regression analyses. We confirmed that the proportional hazards assumption was not violated in both populations by log–log plots and by the inclusion of time-dependent interaction terms for covariates with survival time in the models. All tests were two-tailed, and all analyses were performed using Statistical Package for Social Science Professional software version 20.0 (SPSS, Chicago, IL USA). A two-tailed *P* < 0.05 was considered statistically significant.

## Supplementary information

Supplementary information
