# Supplementary material for: Decreased expression of the thyroid hormone-inactivating enzyme type 3 deiodinase is associated with lower survival rates in breast cancer
Source: Sci Rep. 2020 Aug 17;10:13914. doi: 10.1038/s41598-020-70892-4 (PMC7431418; doi:10.1038/s41598-020-70892-4)
Supplement: Supplementary file 1 — Supplementary information [file 41598_2020_70892_MOESM1_ESM.pdf]

Decreased expression of the thyroid hormone-inactivating enzyme type 3 deiodinase is associated with lower survival rates in breast cancer

Iuri Martin Goemann*, M.D., Ph.D.[1]; Vicente Rodrigues Marczyk[1,5]; Mariana Recamonde-Mendoza, Ph.D.[2,3]; Simone Magagnin Wajner, M.D., Ph.D.;[1,5], Marcia Silveira Graudenz, M.D., Ph.D[4,5] and Ana Luiza Maia*, M.D., Ph.D.[1,5]

Correspondence: Dr. Ana Luiza Maia

Serviço de Endocrinologia

Hospital de Clínicas de Porto Alegre

Rua Ramiro Barcelos, 2350 CEP 90035-003 – Porto Alegre, RS, Brasil.

Phone/Fax: 55- 51- 33310207. E-mail: almaia@ufrgs.br

Supplementary Figure 1

A. ER+ Patients

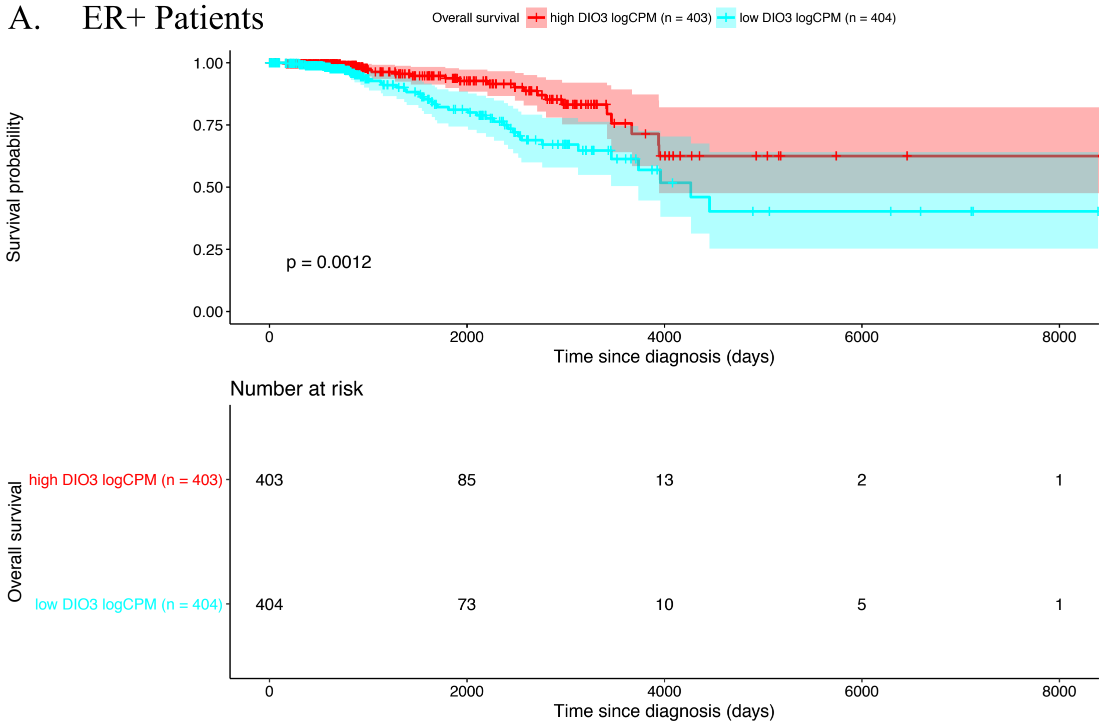

B. ER- Patients

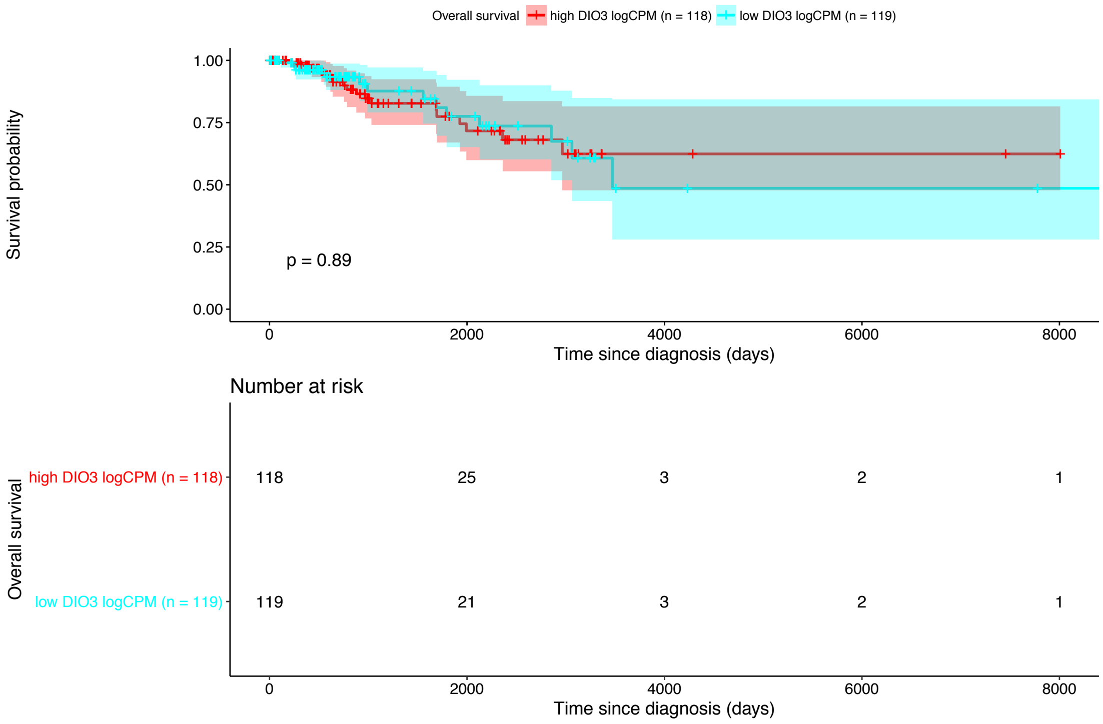

Supplementary Fig. 1 Subgroup analysis of overall survival of patients from the TCGA-BRCA cohort according to *DIO3* mRNA expression, where red lines refer to patients high *DIO3* expression and blue lines refer to patients with low *DIO3* expression. Groups are divided according to the median of *DIO3* expression in the population. (a) Kaplan-Meier plot demonstrates overall survival of patients with ER-positive breast cancer. (b) Kaplan-Meier plot of patients with ER-negative breast cancer. ER = estrogen receptor
